# Knowledge predicts agility but not power: the role of injury prevention awareness in the functional performance of female collegiate athletes

**DOI:** 10.7717/peerj.21327

**Published:** 2026-05-22

**Authors:** Monira I. Aldhahi, Smaher Abdullah Aldawood, Raghad Khalid Alotaibi, Haneen Hamoud Alsahli, Fay Ahmed Faqihi, Rawan Mansour Binsaad, Reem M. Alwhaibi

**Affiliations:** Department of Rehabilitation Sciences, College of Health and Rehabilitation Sciences, Princess Nourah bint Abdulrahman University, Riyadh, Saudi Arabia

**Keywords:** Knowledge, Injury prevention, Sports injury, Prevention program, Female athletes, College athletes

## Abstract

**Background:**

Injury prevention is essential for sustaining athletic performance and minimizing risks. Although knowledge and attitudes toward injury prevention are believed to influence outcomes, their relationship with functional performance remains underexplored. This study aimed to assess the association between injury prevention knowledge, attitudes, and performance metrics among female collegiate athletes.

**Methods:**

Sixty female collegiate athletes were recruited for this study. Injury prevention knowledge and attitudes were assessed using validated questionnaires. Functional performance was evaluated using the Star Excursion Balance Test (SEBT), Countermovement Standing Long Jump (SLJ), Single-Leg Hop (SLH), and Lower Extremity Functional Test (LEFT). Pearson correlations and hierarchical linear regression were used to examine relationships between variables (*p* < 0.05).

**Results:**

The mean knowledge score was 16.3 out of 26 (SD = 4.82, 62.7% of maximum), ranging from 5 to 24. While 51.67% of the respondents showed a positive attitude, 48.33% had a suboptimal attitude. The mean SLH distances (dominant: 128.6 cm; non-dominant: 126.31 cm) exceeded the normative values. LEFT time (160 ± 19 seconds) and Peak Anaerobic Power (2,387.85 W) reflected slower completion and reduced power. SEBT scores were below 94% threshold in all directions. Knowledge was moderately correlated with LEFT (*r* =  − 0.42, *p* = 0.0009), and no significant associations were found between attitude and performance. A hierarchical linear regression analysis showed that training-related variables did not significantly predict LEFT performance. However, the inclusion of injury prevention knowledge significantly improved the model, with knowledge emerging as a significant predictor, explaining an additional 14% of the variance in LEFT performance (*β* = 0.39, *p* = 0.003).

**Conclusions:**

Knowledge of injury prevention is associated with enhanced agility in athletes. However, attitude was not significantly related to the performance outcomes. These insights underscore the importance of integrating targeted injury prevention education into training programs for female athletes.

## Introduction

Female athletes face substantial injury risks, particularly chronic overuse injuries, such as muscle strains and ligament injuries, which result from biomechanical differences, training demands, and sport-specific movement patterns (Baugh et al., 2018; International Olympic Committee Injury Illness Epidemiology Consensus Group et al., 2020; Szukics et al., 2022). While sports participation offers significant physical and psychological benefits, effective injury prevention requires evidence-based interventions that account for gender-specific physiological characteristics and biomechanical demands (Emmonds, Heyward & Jones, 2019). Despite established prevention programs reducing injury incidence by up to 40% in research settings (Hootman, Dick & Agel, 2007; Soomro et al., 2016), translating this knowledge into consistent practice remains challenging, particularly in populations with limited access to professional coaching, injury prevention resources, or prevention-focused education. Additionally, these interventions often show inconsistent effectiveness, partly because they fail to account for gender-specific biomechanical characteristics (Bruinvels et al., 2017) and athlete-level factors such as knowledge and attitudes toward prevention (Emmonds, Heyward & Jones, 2019). Current evidence suggests that knowledge and attitudes influence injury prevention behavior; however, limited research has examined whether this knowledge-behavior pathway translates into measurable improvements in physical performance, which is a critical link for understanding how educational interventions can reduce injury risk.

Saudi Arabia provides a unique context for examining the relationship between knowledge, attitudes, and performance. Recent initiatives promoting female sports participation have expanded access to athletic facilities; however, they have also revealed elevated injury prevalence: 62.2% of female athletes reported sports injuries compared to 32% in Switzerland (Michaud, Renaud & Narring, 2001; Alhomaied et al., 2022). This disparity suggests that access to facilities alone is insufficient without concurrent injury prevention. This creates an opportunity to examine whether existing models of knowledge, attitudes, and physical performance generalize across different sociocultural and resource contexts.

The existing literature supports a conceptual pathway in which injury prevention knowledge shapes attitudes and behaviors (Rees et al., 2022). However, research has largely examined knowledge and attitudes in isolation or explored their relationship with *adherence* to prevention programs, not *objective physical performance outcomes*. This gap is particularly concerning because injury prevention ultimately aims to improve physical capabilities while reducing injury risk; however, we lack empirical evidence demonstrating whether enhanced knowledge translates into measurable improvements in lower extremity performance metrics (balance, power, and agility) that contribute to injury resilience. Furthermore, no studies have examined this knowledge-performance relationship within female athletes in low-resource settings where injury prevention infrastructure is developing, limiting understanding of how interventions can be optimally designed for such populations. Prior literature supports the theory that greater awareness of injury risk and positive attitudes toward prevention can influence not only behavior but also athletic performance through better self-regulation, training adherence, and body awareness (Avedesian et al., 2022). For example, athletes who understand safe landing, deceleration, and cutting mechanics may execute change-of-direction tasks with improved alignment and control, potentially enhancing agility and dynamic balance performance (Geertsema et al., 2021; Martinez, Alexander & Pagnotta, 2024; Gabriel & White, 2024). These performance outcomes were selected because they closely reflect movement qualities associated with injury risk and neuromuscular control. Agility (Lower Extremity Functional Test) and dynamic balance (Star Excursion Balance Test) were selected because they reflect neuromuscular control during cutting and stabilization tasks, which are strongly linked to injury risk (Aldhahi et al., 2026). Lower-limb power was assessed using hop and jump measures (Single-Leg Hop and Countermovement Standing Long Jump), as these outcomes reflect force production and landing control that may be influenced by athletes’ preventive knowledge and training behaviors.

Despite the theoretical support for this relationship, empirical evidence directly linking injury prevention knowledge to objective functional performance outcomes remains limited. Specifically, few studies have examined whether injury prevention knowledge and attitudes are associated with objective lower extremity performance measures, such as agility, balance, and power, particularly among female collegiate athletes. The majority of current literature has focused on the demographic correlates of knowledge and attitudes and has not gone further. No study has empirically tested whether injury prevention knowledge predicts objective lower extremity functional performance outcomes. Second, agility, balance, and power have not been compared within a single study to map whether knowledge selectively benefits tasks requiring cognitive-motor integration over those driven by physiological capacity alone. All prior work has been conducted in Western, resource-rich settings, leaving open the question of whether knowledge-performance relationships observed elsewhere generalize to populations such as female collegiate athletes in Saudi Arabia, where historically limited access to professional coaching and injury-prevention infrastructure may produce distinct patterns of knowledge acquisition, attitude formation, and functional performance. The present study was designed to address all three gaps simultaneously.

This study aimed to comprehensively assess injury prevention knowledge, attitudes, and lower extremity performance capabilities among female college athletes in Saudi Arabia while systematically identifying barriers to implementing evidence-based prevention practices. Specifically, this study examined the associations between injury prevention knowledge and objective lower extremity performance measures (power, agility, and balance) and perceived facilitators and barriers to implementing injury prevention strategies, including resource constraints, coaching availability, cultural factors, and institutional support. We hypothesized that greater injury prevention knowledge correlates with superior lower extremity performance and that modifiable barriers significantly attenuate the knowledge-to-performance translation. By integrating educational, physical performance, and implementation science perspectives within an understudied population, this study will provide theoretical evidence regarding the generalizability of injury prevention models across contexts and actionable insights into context-specific barriers that must be addressed to successfully implement evidence-based interventions for female athletes in Saudi Arabia and similar emerging sports settings. By bridging the gap between educational awareness and objective physical outcomes, this study seeks to inform the development of gender-sensitive, evidence-based injury prevention programs tailored to the needs of female athletes in Saudi Arabia and in similar settings.

## Materials & Methods

### Study design & Participants

A cross-sectional study was conducted on female collegiate athletes (*i.e.,* athletes currently enrolled in a university program and representing a university team or participating in organized collegiate-level training/competition). An observational cross-sectional design was selected for two primary reasons. First, the relationship between injury-prevention knowledge and performance in this population has not previously been examined; therefore, establishing the presence, direction, and magnitude of this association is necessary before experimental manipulation can be justified. Second, within the context of female collegiate athletes in Saudi Arabia—where injury-prevention systems are still developing—mapping current levels of knowledge and performance represents an appropriate initial step. Accordingly, a cross-sectional design is suitable for this stage of evidence development.

Participants were eligible for inclusion if they met the following criteria: (1) female college students aged between 18 and 35 years; (2) currently engaged in regular athletic training or participation in organized sports at the collegiate level with a minimum of one year of experience in their respective sports; (3) free from any acute musculoskeletal injuries at the time of testing; (4) able to perform lower extremity performance tests without restriction; and (5) willing to provide informed consent and complete all components of the study protocol. Participants were excluded if they reported any recent injury (within the last six months) that limited performance, pregnancy, or any chronic medical condition that might have influenced the study outcomes. Health status and a history of injury were confirmed during the initial screening process as part of the inclusion criteria. Collegiate status, health status, and injury history were verified during screening using a structured eligibility checklist and a brief standardized interview. Participants confirmed their current university enrollment and collegiate-level sports participation and reported their recent injury history and medical conditions. Where applicable, the athletes were asked to provide team/club affiliation and a training context to confirm eligibility. Participants with uncontrolled diabetes or hypertension were excluded from the study. After fulfilling the inclusion criteria, the participants’ consent forms were obtained prior to their entry into the study.

A priori sample size calculation was performed using G*Power (version 3.1.9.7; Heinrich Heine University Düsseldorf, Düsseldorf, Germany) (Faul et al., 2009). Assuming a moderate effect size (Cohen’s *f*^2^ = 0.35), alpha level of 0.05, statistical power of 0.80, and five predictors in the regression model, the minimum required sample size was estimated to be 53 participants. To account for potentially incomplete data, recruitment remained open throughout the data collection period until the target sample size was achieved.

Given the limited number of female athletes in the university setting, a convenience sampling method was employed to recruit participants for voluntary participation in this study. Invitations to participate in the study were distributed broadly through official channels, including the Saudi Sports Federations and the Ministry of Sports. This approach was selected because of feasibility constraints and the limited availability of eligible female collegiate athletes across sports, while leveraging centralized channels that reach university-affiliated athletes. Participants were recruited using a non-probability convenience sampling method. Although we did not define a fixed initial pool size, participation was open to all eligible female collegiate athletes who responded to the invitation during the data collection period.

**Figure 1:**
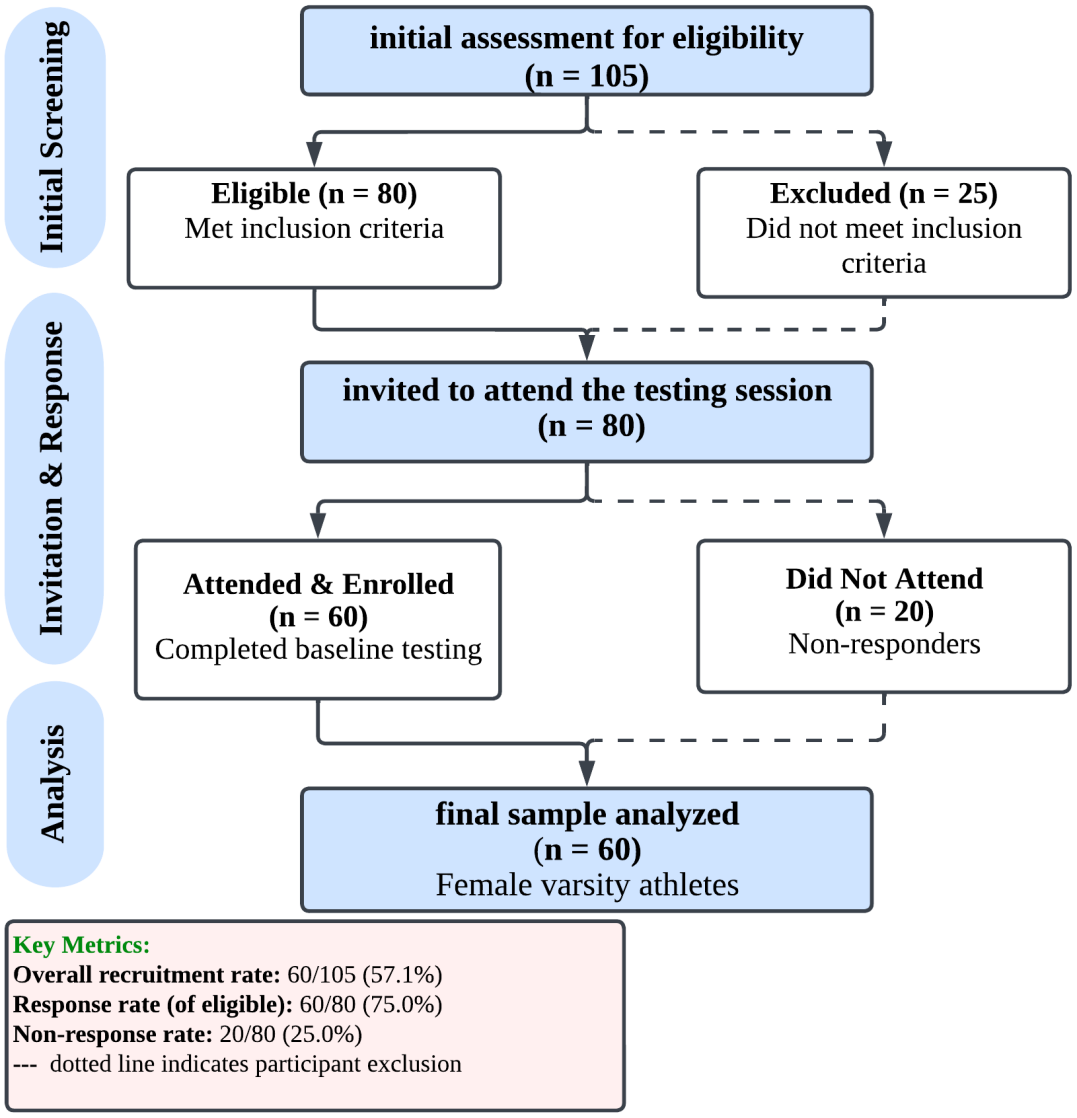
Study recruitment flowchart. Illustration of overall recruitment metrics, including eligibility assessment, group allocation, and participant retention throughout the study.

Data were collected during the off-season, following the completion of the competitive sports season. Sixty athletes completed the study assessments and were included in the final analysis. Of 105 individuals initially assessed for eligibility, 25 did not meet inclusion criteria. The remaining 80 eligible participants received formal invitations to attend the baseline testing sessions. Sixty participants (75% response rate) attended and completed the assessment battery, while 20 eligible participants (25%) did not respond to the invitation or attend the scheduled sessions. The final analysis included all 60 participants. All participants included in the final analysis (*N* = 60) completed the assessment battery in full, with no missing data. Participants were not excluded from the specific analyses based on fatigue or performance issues (Fig. 1).

This study was conducted in accordance with the Declaration of Helsinki and was reviewed and approved by the Institutional Review Board Committee of Princess Nourah bint Abdulrahman University (Approval Number: 24-0937, dated December 26, 2024). Written informed consent was obtained before participation, and participation was voluntary; participants were allowed to withdraw at any time without any consequences.

### Procedures

Participants were screened based on the established inclusion and exclusion criteria. Initially, the participants completed a structured questionnaire to gather demographic information and assess their knowledge, attitudes, and perceived barriers or facilitators toward injury prevention programs. The questionnaire was administered electronically to ensure accessibility and ease of completion.

Subsequently, the participants underwent a battery of lower extremity performance tests to evaluate power, agility, and balance. These tests included the Lower Extremity Functional Test (LEFT) to measure agility through eight movement tasks and the Single-Leg Hop (SLH) for Distance Test to predict injury risk based on unilateral jump performance. Additionally, the Star Excursion Balance Test (SEBT) was conducted to evaluate dynamic balance and stability, while the Countermovement Standing Long Jump (SLJ) Test was used to determine peak power. Prior to data collection, participants were instructed to refrain from strenuous physical activity for at least 24 h prior to their testing session and to avoid any competitive match play within 48 h of assessment. Participants who had been involved in formal competition within this window were rescheduled to ensure comparable recovery status across the sample. On the day of testing, all participants completed a standardized 10-minute progressive warm-up protocol (5 min of light jogging followed by 5 min of dynamic stretching targeting the lower extremities) before commencing the performance battery.

To further control for pre-testing training load, participants were provided with a familiarization session to ensure understanding of the testing procedures and to reduce potential learning effects during formal assessment. All participants completed the assessment procedures in a fixed and identical order. A standardized 10-minute rest interval was provided between consecutive tests to minimize fatigue. The testing protocol was consistently applied to all participants to enhance reproducibility.

### Outcome measures

#### Knowledge and attitude

The questionnaire was administered in Arabic and English and consisted of four sections. The first section of the questionnaire is the demographic section. This section collected data from participants regarding their age, gender, the team sport they play, any injuries they have sustained in recent years, their current health status, and their educational program. Second section adopted from questionnaire titled “Knowledge of Sport Injury Prevention and Management” (Wang, Lin & Huang, 2012). Knowledge of sports injury prevention was assessed using a 26-item multiple-choice questionnaire. Items were organized into five domains: injury identification domain (4-itmes), acute injury management domain (4-itmes), support and immobilization (4-itmes), emergency response (8-itmes), and injury prevention program (6-itmes). Each item had one correct answer; correct responses were scored as 1 and incorrect responses as 0, yielding a total knowledge score ranging from 0 to 26, with higher scores indicating greater knowledge. The knowledge score was treated as a continuous variable. To ensure the robustness of the findings, knowledge was included in the regression models as a continuous variable.

The third section focuses on attitude and is based on two established and validated surveys from previous research studies. One section of the questionnaire was adapted from a study on injury behaviors and attitudes, originally designed to evaluate high school athletes’ views on concussion behaviors in high-risk sports (Kurowski et al., 2014). The other section draws from a study, a sports injury prevention survey, which focused on female soccer players’ beliefs and attitudes regarding injury prevention (Geertsema et al., 2021). These questions collectively assessed attitudes towards injury and injury prevention strategies using a validated 14-item scale. Responses were recorded on a 5-point Likert scale ranging from “Never/Not Important” to “Always/Very Important.” with 1 being the lowest score and 5 the highest score per question. Each response indicative of a negative attitude was assigned 0 points, while those reflecting a positive attitude were assigned 1 point. The total attitude score ranged from 0 to 14, and the scores were converted to percentiles.

For questionnaires administered in a language other than English, a standardized translation and back-translation procedure was employed. The original English version was translated into Arabic by a bilingual researcher with subject-matter expertise. An independent bilingual translator, blinded to the original version, then back-translated the questionnaire into English. The original and back-translated versions were compared, and discrepancies were resolved through consensus to ensure semantic and conceptual equivalence. Cultural adaptation focused on maintaining conceptual meaning rather than literal translation (Eremenco, Cella & Arnold, 2005). The questionnaire was subsequently pilot tested in a sample of 20 participants representative of the study population to assess comprehension and completion time. Feedback from the pilot participants indicated that all items were understandable and relevant, and no further modifications were required.

The content validity of the questionnaires was evaluated using an expert review approach (Polit, Beck & Owen, 2007). An expert panel comprising two specialists in sports physical therapy and rehabilitation science independently reviewed the questionnaire items. The experts assessed each item for relevance, representativeness, and clarity concerning the study objectives and construct of injury-related knowledge. Items were rated on a (4-point Likert scale ranging from “not relevant” to “highly relevant”). Based on expert feedback, items with low relevance ratings were revised or removed, and wording adjustments were made to improve clarity and coverage. Consensus discussions were used to resolve discrepancies and ensure that all items adequately reflected the intended content domain. For items with a content validity index (CVI) of > or = 0.80, high content validity was considered, and items with CVI > 0.80 were maintained in the questionnaire. The internal consistency of the knowledge questionnaire was acceptable (Cronbach’s α = 0.79), and attitude was high, with a Cronbach’s alpha coefficient of 0.86, indicating good reliability. The item–total correlations ranged from 0.33 to 0.60, and the item difficulty indices ranged from 0.38 to 0.79. No items were removed.

The fourth and final section focused on the facilitators and barriers to injury prevention programs. In this section, athletes were asked to identify factors that facilitate or hinder the implementation of injury prevention programs. This part of the survey was adapted from a questionnaire developed in a previous study (Geertsema et al., 2021).

#### Lower extremity performance measure

##### Lower Extremity Functional Test (LEFT).

The lower extremity functional test is commonly used to assess the risk of injury and includes eight agility tasks (Brumitt et al., 2016; Shi et al., 2021). The test was performed on a diamond-shaped course without rest intervals. The LEFT consists of eight components: forward run, backward run, side shuffle, carioca, figure 8 run, 45° cuts, 90° cuts, forward run, and backward run. The test-retest reliability of the LEFT is considered excellent, with values ranging from 0.95 to 0.97 (Tabor et al., 2002; Shi et al., 2021). It is known to predict the risk of injury and was used to categorize participants based on their performance, with an average completion time of approximately 135 s (range: 120–150 s) for women (Brumitt et al., 2016; Shi et al., 2021). Those who showed a longer duration score were placed in the high-risk group, while those who achieved a shorter time to complete the test were placed in the low-risk group. The intraclass correlation coefficients (ICCs) were high (ICC = 0.97) (Tabor et al., 2002), and the test showed significant accuracy in discriminating between injured and uninjured players (AUC 0.908, 95% CI [1.126–1.336], *p* = 0.001) (Mohammadi et al., 2024).

##### Countermovement Standing Long Jump (SLJ).

Before testing, the standing reach distance of each participant was measured and recorded. The participants began the test barefoot, standing with their feet shoulder-width apart, positioned just behind a designated starting line on a non-slip hard surface. A vertical jump tester was used for measurement. The participants were instructed to perform a countermovement jump by bending their knees to a self-selected depth and using a coordinated arm swing to maximize propulsion. Each jump was executed with a simultaneous two-foot takeoff and required a controlled two-foot landing.

Each participant completed three valid trials, and the longest jump distance was recorded as their final score. The standing reach distance was then subtracted from this value to determine the participant’s net jump performance (Sayers et al., 1999).

The SLJ was used to assess lower extremity power performance and was associated with the risk of injury (Pedley et al., 2020). The test is frequently used to assess peak anaerobic power, which is determined from measurements of force and horizontal distance jumped (Markovic & Jaric, 2007). The equation from a previous study was used to measure peak anaerobic power: squat jump peak power (W) = 60.7  × height (cm) + 45.3  × body mass (kg)—2055 (Sayers et al., 1999). Vertical jump height was calculated using a vertical jump meter. Improvements in jump height have been associated with improved strength and athletic performance. It is widely administered during rehabilitation phases and performance enhancement training (Nuzzo, Anning & Scharfenberg, 2011). The test-retest trial had good reliability (ICC = 0.97) (Buckthorpe, Morris & Folland, 2012). The structural validity of the SLJ was evaluated using factor analysis. The countermovement standing long jump demonstrated the highest loading on the explosive power factor (*r* = 0.87), confirming its factorial validity (Markovic et al., 2004).

##### The Single-Leg Hop (SLH).

It is a widely used functional performance measure that assesses lower extremity strength, neuromuscular control, balance, and muscle power. It requires an individual to hop as far as possible and land safely on the same leg. On the Jump Measuring Mat, the participants hopped forward on one leg for the maximum distance, aiming to land with stability and control. Both dominant and non-dominant legs were assessed to identify any functional asymmetries. Recent studies have demonstrated excellent test-retest reliability of the SLH test. The test has been reported to have an intraclass correlation coefficients (ICC) ranging from 0.98 to 1.00 (Negrete et al., 2021). Studies have indicated that deficits or asymmetries observed during single-leg hop testing are associated with an increased susceptibility to lower limb injuries (Hegedus et al., 2015). Significant positive correlations were found between the LSI of the single-limb hop distance and the LSI of the peak extension torque at 120°/s (*p* = 0.044, *r* = 0.37), peak extension torque at 180°/s (*p* = 0.042, *r* = 0.38), and peak flexion torque at 180°/s (*P* = 0.043, *r* =  − 0.38) (Xergia, Pappas & Georgoulis, 2015).

##### Star Excursion Balance Test (SEBT).

This test is widely used to measure lower extremity balance (dynamic and static) and stability. The test features lines arranged in a grid that extend from a center point positioned 45° to each other. It requires that one leg be used to reach the anterior, posterior, anteromedial, anterolateral, posteromedial, and posterolateral while standing on the other limb. Failure in the SEBT is associated with a higher incidence of lower limb injury (Plisky et al., 2006; Stiffler et al., 2017). Differences in SEBT performance often reflect underlying biomechanical deficiencies that predispose individuals to injury (Gribble, Hertel & Plisky, 2012). The SEBT exhibits good-to-excellent test-retest reliability (interrater reliability = 0.89–0.93) (Gribble, Hertel & Plisky, 2012), and the ICC for intra-rater reliability ranges from 0.85 to 0.91 (Plisky et al., 2009). There were very strong correlations (Pearson *r* > 0.96) between the observer and motion capture measurements, which confirmed high concurrent validity (Kanko et al., 2019). The SEBT can predict the risk of lower extremity injuries according to predictive validity, particularly when evaluating side-to-side asymmetries in the anterior direction (Stiffler et al., 2017).

### Data analysis

The raw data were screened for completeness by examining all variables using frequency tables and summary statistics. The data were then evaluated for outliers and implausible values using descriptive statistics (means, standard deviations, minimums, and maximums) and graphical methods, including histograms and boxplots. Values outside the expected or physiologically plausible ranges were flagged and verified against the original data entries. Logical consistency checks were performed using cross-tabulations and conditional screening to identify any incompatible or contradictory responses. Identified inconsistencies were reviewed and corrected where necessary before analysis. The Shapiro–Wilk and Kolmogorov–Smirnov tests were conducted to assess whether the data followed a normal distribution. These tests evaluated the normality of the data distribution, ensuring that appropriate statistical methods were used. Descriptive statistics are reported as mean ± standard deviation (SD) for continuous variables and frequency (n) with percentages (%) for categorical variables. All questionnaire items were numerically coded in Stata before analysis. Knowledge items were coded as 1 for correct responses and 0 for incorrect responses, and domain and total knowledge scores were computed. Pearson correlation coefficients were calculated to examine the relationship between knowledge, attitude, and injury risk performance measures (SLJ, SLH, LEFT, and SEBT). Regression assumptions, including linearity, homoscedasticity, normality of residuals, and multicollinearity (VIF), were evaluated prior to analysis. Hierarchical linear regression was used to examine the association between injury-prevention knowledge and lower extremity performance. Covariates (number of sports types, training duration, frequency, and history of injury) were entered in the first block based on theoretical relevance. Knowledge was entered in the second block to determine its unique contribution beyond the covariates. All data were analyzed using Stata version 17 (Stata Corp LP, College Station, TX, USA). Statistical significance was set at *p* < 0.05.

## Results

### Demographic and training characteristics of participants

The study included 60 female athletes with a mean age of 20 years (±3 years; range, 18–35 years) and a mean body mass index (BMI) of 21.62 kg/m^2^ (Table 1). The majority of participants were non-smokers (93.33%). Regarding educational background, most participants were undergraduates (90%), while 2% had completed high school, and 8% were graduate students. The athletes competed in multiple sports, with basketball being the most common (*n* = 25, 41.67%), followed by volleyball (*n* = 21, 35%) and football (*n* = 16, 26.67%). Many athletes participated in multiple sports (*M* = 2 sports). The mean training duration was 174 min per session (SD = 67.15), with participants training four days per week on average (SD = 1, range: 1–6 days). Approximately 30% (*n* = 18) of the participants reported a history of prior injury. Limb dominance was predominantly right-leg (88.33%, *n* = 53) (Table 1). All 60 participants were varsity-level athletes competing at the university level.

**Table 1:** Demographic and training characteristics of participants.

**Variables**		**Mean**	**SD**
Age (Year)		20	3
Gender (Female, n (%) ^*^		60	100
Smoking Status n (%) ^*^	Smoking	2	3.33
	Not smoking	56	93.33
	Quit smoking	2	3.33
Height (cm)		158.01	6.11
Weight (kg)		53.99	8.26
Education Categories, n (%) ^*^	High school	1	2
	Undergraduate	54	90
	Graduate	5	8
	Other	0	0
Type of sports, n (%) ^*^^,^^a^	Football	16	26.67
	Rugby	4	6.67
	Basketball	25	41.67
	Volleyball	21	35
	Weightlifting	13	21.67
	Swimming	7	11.67
	Other (Tennis/badminton/Dodgeball/Taekwondo/judo/kara)	22	36.67
History of prior injury, n (%) ^*^		18	30
Training frequency (Day/week)		4	1
Sports experiences (Years)		4	3
Number of sports		2	1
Training duration (min)		174	67.15
Dominant leg, n (%) ^*^	Left	7 (11.67)	
	Right	53 (88.33)	

**Notes.**

* Denotes data are presented as frequency (n), and percentage (%). SD denotes standard deviation.

a participants select multiple responses and denominator was the total respondents not the total sports selections.

### Knowledge of injury prevention

Overall, knowledge of injury prevention was moderate, with participants achieving a mean score of 16.3 out of 26 (SD = 4.82, 62.7% of maximum). Scores ranged from 5 to 24, showing that while no participant achieved the maximum score of 26, the lowest-scoring participant still demonstrated some baseline knowledge. Domain-level analysis revealed variability in knowledge across the five injury prevention domains. The mean scores for injury identification (Domain 1) were relatively low (1.43 ± 0.83; range 0–3), indicating limited recognition of basic injury types. Scores were higher for acute injury management (Domain 2; 2.92 ± 1.03; range 0–4) and support and immobilization (Domain 3; 1.88 ± 0.92; range 0–3). Participants demonstrated the highest knowledge in the emergency response domain (Domain 4; 4.55 ± 1.64; range 0–7), followed by the injury prevention program (Domain 5; 3.85 ± 1.33; range 1–6). Overall, knowledge was stronger in emergency and injury prevention program-related topics, while injury identification and basic support techniques showed comparatively lower scores (Table 2).

**Table 2:** Percent-of-maximum knowledge scores across injury-prevention domains.

**Domain**	**Mean ± SD**	**Max**	**% of Maximum**
Domain 1 –Injury identification	1.43 ± 0.83	3	47.8%
Domain 2 –Acute injury management	2.92 ± 1.03	4	72.9%
Domain 3 –Support & immobilization	1.88 ± 0.92	3	62.8%
Domain 4 –Emergency response	4.55 ± 1.64	7	65.0%
Domain 5 –Injury prevention program	3.85 ± 1.33	6	64.2%

**Notes.**

Values expressed as Percent-of-maximum scores (%) were calculated as (mean domain score ÷ maximum possible domain score) ×10.

### Attitude toward injury prevention

Participants demonstrated predominantly positive attitudes toward injury prevention (mean = 9.25, SD = 3.35). The majority of participants (*n* = 31, 51.67%) demonstrated a positive attitude, indicating strong awareness and acceptance of injury prevention strategies. A total of 29 participants (48.33%) exhibited suboptimal attitudes, suggesting a need for further education and reinforcement of injury prevention measures.

**Figure 2:**
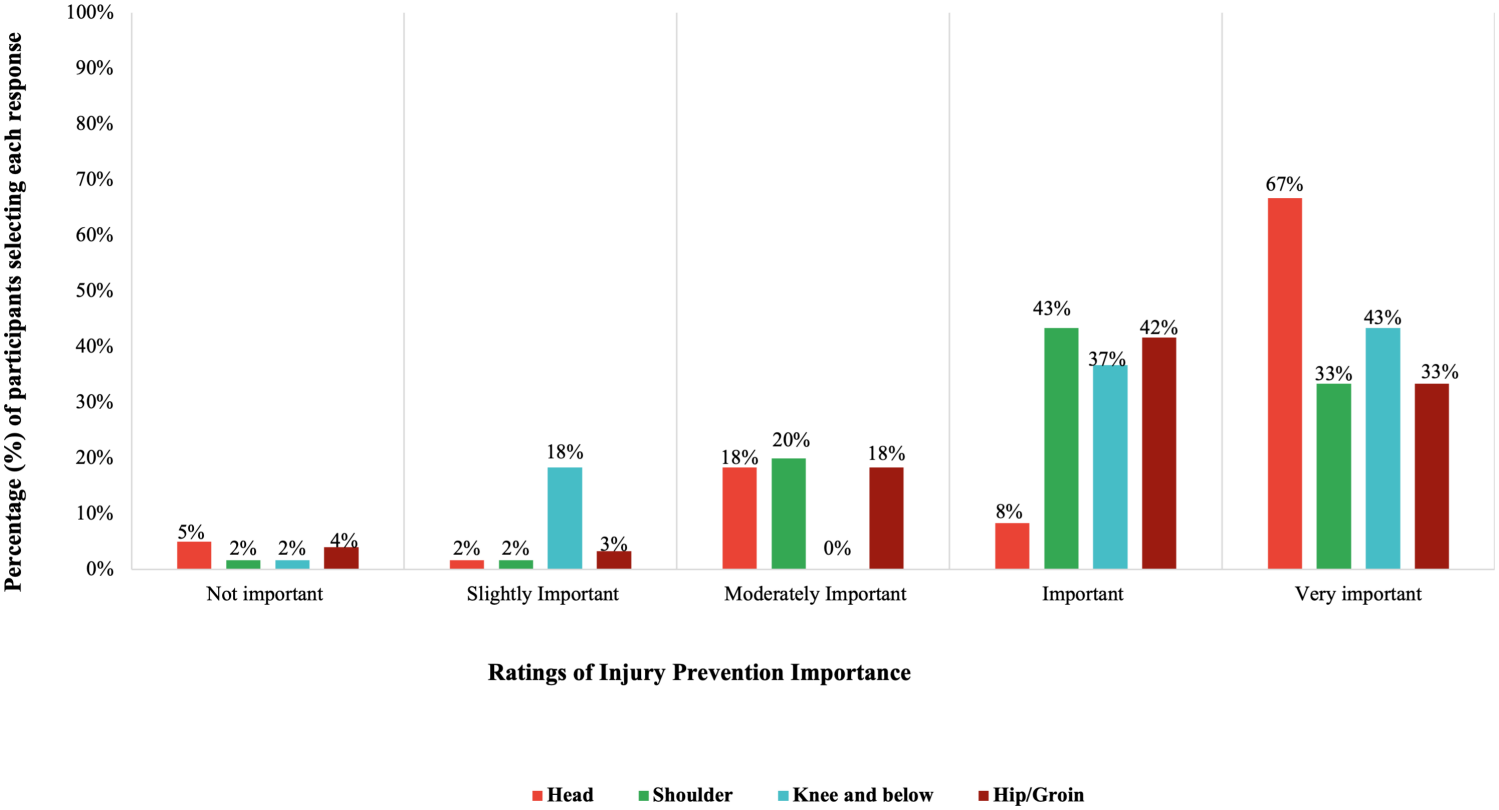
Illustrates the percentage distribution of participants perceived importance of preventing injuries across different body regions (*N* = 60). Participants’ attitude to the prevention of injury in different body locations. Each bar represents the percentage of participants rating the importance of injury prevention for specific body regions, including the head, shoulder, knee and below, and hip/groin areas. The responses were categorized on a five-point Likert scale ranging from “Not Important to Very Important”.

A descriptive analysis was conducted to assess the participants’ attitudes toward injury prevention, as summarized in Table S1. Of the 14 attitude items, higher scores (*i.e.,* scores ranging from 3–4) were reported for eight items. When asked about the importance of injury prevention, 71.67% of participants rated it as “very important,” yet only 5% reported being “extremely informed” about available prevention programs. Overall, 53.33% of the participants expressed positive attitudes toward injury prevention measures. Figure 2 provides a comparative visualization of participants perceived importance of preventing injuries across specific body regions. Figure 2 highlights body region–specific priorities. Participants consistently rated injury prevention as “important” or “very important.” Head injury prevention received the highest importance rating (67% “very important”), followed by shoulder (43%) and hip/groin (42%) injury prevention. Notably, 33.33% (*n* = 20) indicated that they would immediately inform healthcare professionals about injuries, even if it meant missing matches, which is a protective behavior. However, 16.67% (*n* = 10) endorsed playing through injuries for team success, indicating concerning attitudes toward injury prioritization.

### Risk of injury functional performance assessment

#### Star Excursion Balance Test (SEBT)

Overall, the reach distances were comparable between the dominant and non-dominant legs, with minor variations across specific directions (Table 3). The posterior direction showed a slightly greater reach in the non-dominant leg (70.27 ± 12.05 cm) than in the dominant leg (68.88 ± 13.12 cm). Similarly, in the posteromedial direction, the non-dominant leg (66.75 ± 12.13 cm) had a slightly higher reach than the dominant leg (64.75 ± 12.44 cm). In contrast, the medial direction showed a greater reach in the dominant leg (63.15 ± 10.77 cm) than in the non-dominant leg (61.18 ± 12.04 cm). The results in Table 4 indicate that the relative reach distances in all directions were below the threshold, which was 94% for women, based on the normative data of a previous study. The posteromedial (78.10 ± 16.14%) and medial (75.07 ± 15.87%) directions showed lower reach scores (<80%), which may indicate reduced neuromuscular control and postural stability.

**Table 3:** Demonstrate the result of Star Excursion Balance Test (SEBT) and Single-Leg Hop Test.

**Variables**	**Dominant Leg**	**Nondominant Leg**
	**Mean ± SD**	**Mean ± SD**
Relative Anterior (%)	88.29 ± 12.362	87.39 ± 13.16
Relative Anterolateral (%)	83.058 ± 17.02	83.21 ± 14.25
Relative Lateral (%)	80.01 ± 14.93	80.18 ± 14.78
Relative Posterolateral (%)	81.86 ± 16.74	84.34 ± 15.45
Relative Posterior (%)	84.17 ± 16.22	87.25 ± 17.71
Relative Posteromedial (%)	78.10 ± 16.14	81.38 ± 15.64
Relative medial (%)	75.07 ± 15.87	74.22 ± 15.56
Relative Anteromedial (%)	81.52 ± 17.86	81.51 ± 17.45
Anterior (cm)	72.70 ± 9.49	72.65 ± 9.92
Anterolateral cm)	68.64 ± 12.31	68.433 ± 10.238
Lateral (cm)	65.06 ± 11.75	64.91 ± 11.32
Posterolateral cm)	67.72 ± 12.96	68.71 ± 11.89
Posterior (cm)	68.88 ± 13.12	70.27 ± 12.05
Posteromedial (cm)	64.75 ± 12.438	66.75 ± 12.13
Medial (cm)	63.15 ± 10.77	61.18 ± 12.04
Anteromedial (cm)	67.91 ± 10.29	66.96 ± 11.87
Composite SEBT (%)^*^	81.51 ± 12	82.44 ± 12.50
Single Leg Hop (cm)	128.6 ± 22.69	126.31 ± 26.04

**Notes.**

* denotes that the composite SEBT calculated based on average of the reach distances of the dominant leg in all directions normalized to leg length and expressed as a percentage.

**Table 4:** Characteristic of LEFT and countermovement SLJ performance.

**Variables**	**Mean ± SD**		**Min**	**Max**
LEFT (sec)	160 ± 19		122	221
SLJ Hight (cm)	32.90 ± 7.62		14	53
PAP (W)	2,387.85 ± 522		1,333.4	3,544

**Notes.**

Abbreviations LEFT Lower Extremity Functional Test PAP Peak Anaerobic Power SLJ Countermovement Standing Long Jump W Watts

#### Single Leg Hop Test (SLH)

In the SLH test, the results indicated a slightly greater hop distance in the dominant leg (128.6 ± 22.69 cm) than in the non-dominant leg (126.31 ± 26.04 cm). Normative data indicate that healthy female athletes typically achieve hop distances averaging 121 cm. The participants in this study demonstrated mean hop distances of 128.6 cm (dominant leg) and 126.31 cm (non-dominant leg), exceeding the normative averages.

#### Lower extremity functional test (LEFT) and countermovement SLJ

The normative values of LEFT (in seconds) for female athletes typically range around 120 s, whereas the participants in this study recorded a mean time of 160 ± 19 s. This longer completion time indicates lower agility compared to standard athletic norms.

Table 4 presents the SLJ measures peak anaerobic power (PAP, in Watts) and jump height (in cm). Female athletes typically achieve jump heights between 30 and 40 cm. In this study, the participants’ mean jump height of 32.9 cm fell within the expected range but was slightly below the elite standards. This suggests a moderate level of lower body power. Compared to the normative PAP value for female athletes (2,993.7 W), the participants’ mean PAP (2,387.85 W) was considerably lower.

### Relationship between knowledge, attitude, and injury risk measures

Table 5 presents the Pearson correlation coefficients between knowledge and various injury risk performance measures, including LEFT, PAP, SLJ, and SLH. Knowledge and LEFT (s) showed a significant moderate negative correlation (*r* =  − 0.42, *p* = 0.0009). Negative correlations were observed between knowledge and PAP (*r* =  − 0.07) and SLJ (*r* =  − 0.18), although these were not statistically significant. Although participants reported generally positive attitudes toward injury prevention across all body regions (Fig. 2), these attitudes were not significantly associated with any functional performance outcomes, including agility, balance, or power measures. Consequently, attitude variables were not retained in subsequent regression models, and only predictors demonstrating significant univariate associations were carried forward to multivariable analyses.

**Table 5:** Matrix of pearson correlation between knowledge, attitude and injury risk performance measures.

**Variables**	**Knowledge**	**Attitude**	**LEFT**	**PAP**	**SLJ**	**SLH**	**SEBT**
**Knowledge**	1.0						
**Attitude**	0.08	1					
**LEFT**	−0.42^*^	0.03	1.0				
**PAP**	−0.07	0.16	− 0.30^**^	1.0			
**SLJ**	−0.18	0.22	− 0.39^**^	0.71^**^	1.0		
**SLH**	−0.20	−0.15	− 0.41^**^	0.16	0.44^**^	1.0	
**SBET (%)**	0.15	0.02	− 0.26^*^	0.06	0.24	0.41^**^	1.0

**Notes.**

Abbreviations LEFT Lower Extremity Functional Test PAP Peak Anaerobic Power SLJ Countermovement Standing Long Jump SLH Single-Leg Hop SEBT Star Excursion Balance Test

* Denotes *p*-value <0.05.

** Denotes *p*-value ≤0.001.

### Association between knowledge toward injury prevention and LEFT

A hierarchical linear regression analysis was conducted to examine the association between injury-prevention knowledge and LEFT performance (Table 6). In Model 1, variables including number of sport types, history of injury, training duration, and training frequency were entered. This model was not statistically significant and explained only 8% of the variance in LEFT performance (*R*^2^ = 0.07, *F* = 1.06, *p* = 0.38). However, the inclusion of knowledge in Model 2 yielded a significant overall model (*p* = 0.003), with knowledge being the only significant predictor in the final model, resulting in a 14% increase in explained variance (Δ*R*^2^ = 0.14). The overall model explained 21% of the variance in LEFT performance (*R*^2^ = 0.21, *F* = 9.26, *p* = 0.003). Knowledge emerged as a significant positive predictor of LEFT (*β* = 0.39, *B* = 1.57, 95% CI [0.57–2.62]), indicating that higher knowledge scores were associated with better LEFT performance.

**Table 6:** Hierarchical regression analysis to the LEFT as dependent variable.

**Model**	**Predictors**	**Coefficients** ^a^							**R** ^**2**^	Δ**R**^**2**^	**F**
		*β*	**B**	**SE**	**t**	***p*** **-value**	**95% CI**				
							**Lower**	**Upper**			
Model 1^b^	Constant	–	176.49	9.81	17.99	0.000	156.83	196.16	0.07	0.07	1.06 *P* = 0.38
	Number of Sports type	−0.12	−3.08	3.10	−0.99	0.32	−9.31	3.14			
	History of injury	0.06	0.89	5.58	0.16	0.87	−10.29	12.08			
	Training frequency (time/week)	−0.21	−3.09	2.34	−1.32	0.19	−7.79	1.60			
	Training duration ( min)	0.006	0.003	0.03	0.08	0.93	−0.07	0.08			
Model 2^c^	Constant	–	147.54	13.20	11.18	<0.001	121.07	174.00	0.21	0.14	9.26 *P* = 0.003
	Number of Sports type	−0.17	−3.78	2.90	−1.30	0.19	−9.61	2.04			
	History of injury	−0.05	−2.37	5.31	−0.45	0.65	−13.03	8.28			
	Training frequency (time/week)	−0.06	−0.99	2.29	−0.43	0.66	−5.59	3.60			
	Training duration ( min)	−0.03	−0.01	0.03	−0.29	0.77	−0.08	0.06			
	Knowledge	0.39	1.57	0 .51	3.04	0.004	0.53	2.60			
	Mean VIF	1.18									

**Notes.**

a Dependent Variable: LEFT.

b (Covariates only): Includes number of sports type, history of injury, training duration, and training frequency.

c Model 2 (Full Model): Includes all Model 1 predictors plus knowledge of risk of injury.

*N* = 60.

LEFT dependent variable *β* standardized regression coefficient B unstandardized regression coefficient SE standard error CI confidence interval VIF Variance Inflation Factor (Mean VIF = 1.18, indicating no multicollinearity concerns)

### Participants’ perceived facilitators and barriers to the implementation of injury prevention measures

The participants identified several facilitators and barriers to successfully implementing injury prevention measures (Table 7). Athletes’ motivation (91.67%) and supportive attitudes from coaches (83.33%) were perceived as strong facilitators.

**Table 7:** Participants perceived facilitators/barriers to the implementation of injury prevention program. Data are presented as frequency (n), and percentage (%).

**Items**	**Facilitators**	**Barriers**
	**N (%)**	**N (%)**
Short training program (0–15 min)	46 (76.67)	14 (23.33)
Long training program (more than 15 min)	41 (68.33)	19 (31.67)
Too many matches in a short period of time	21 (35)	39 (65)
Attitude from the coach	50 (83.33)	10 (16.67)
Education of the athletes and coaches regarding injury prevention	56 (93.33)	4 (6.67)
Time constraints	17 (28.33)	43 (71.67)
Free and good equipment for injury prevention	56 (93.33)	4 (6.67)
Improvement in performance	56 (93.33)	4 (6.67)
Trained medical staff (*e.g.*, athletic therapist, sports psychologist, dietician)	57 (95)	3 (5)
Athletic motivation	55 (91.67)	5 (8.33)

The most frequently reported facilitators included trained medical staff (95%), education of athletes and coaches (93.33%), and free and good equipment for injury prevention (93.33%) and performance improvement (93.33%). The most significant barriers identified were time constraints (71.67%) and too many matches in a short period (65%), both of which could limit the effectiveness of injury prevention programs in the future.

## Discussion

The key findings of this study provide important insights into the knowledge, attitudes, and lower extremity performance of female college athletes in injury prevention. The participants demonstrated a moderate level of knowledge, indicating limited knowledge. Most showed a positive attitude toward injury prevention, indicating strong awareness and acceptance of injury prevention strategies; however, gaps persisted in understanding the structured programs. The body-region-specific patterns extend the attitudinal findings reported by revealing how athletes translate general injury prevention attitudes into practical prevention priorities. The greater emphasis placed on preventing head and lower limb injuries may reflect athletes perceived severity, functional consequences, and potential impact on participation associated with these injuries. In contrast, the relatively lower prioritization of shoulder injury prevention may suggest differences in perceived risk or familiarity with preventive strategies for upper limb injuries. Performance findings revealed above-average lower extremity power in the SLH test compared with the general female athlete population. In contrast, the SEBT results showed comparable reach distances between the dominant and non-dominant legs, with all directions falling below the threshold, suggesting balance deficits. Vertical jump scores indicated moderate lower-body power relative to normative PAP values, indicating reduced anaerobic capacity. Additionally, longer completion times on the LEFT suggested lower agility compared with standard values.

A significant moderate negative correlation was observed between knowledge and LEFT performance, with additional negative associations between PAP, SLH, and SLJ. Attitude showed no significant correlation with any of the injury risk variables. The low knowledge scores in 45% of the participants are concerning, as limited awareness can increase the risk of injuries. This finding is inconsistent with previous research, which has highlighted that elite female footballers showed a good level of knowledge of specific injury prevention strategies, and players demonstrated a positive attitude toward injury prevention exercises, reflected in both their favorable perceptions and their reported high levels of adherence (Geertsema et al., 2021). Consistent with the current results, previous studies have linked poor knowledge to higher injury susceptibility, often due to improper training techniques or inadequate implementation of preventive measures (Wang, Lin & Huang, 2012; Almaawi et al., 2020). This concern is particularly pronounced among female athletes, given their anatomical and biomechanical predisposition to injury.

Moreover, this study showed suboptimal dynamic balance performance in female athletes. A low value was observed in the posteromedial direction, suggesting possible neuromuscular control or reduced postural stability in specific motion planes. Although the anterior and lateral directions yielded relatively higher scores, they did not meet the expected cut-off, indicating widespread balance limitations. Relative reach distances in all directions fell below the threshold values, suggesting an elevated risk of lower extremity injuries. Furthermore, previous studies have shown that athletes in dynamic sports generally exhibit superior postural stability compared to those in less dynamic disciplines (Berry et al., 2015). However, our findings showed no consistent advantage for dynamic sports athletes in any movement direction. Interestingly, the non-dominant limb showed better performance in the posterior and posterolateral directions, whereas the dominant limb excelled in the posteromedial direction, consistent with previous reports of no significant influence of limb dominance on SEBT performance (Cug et al., 2016). The small differences in the reach distances between limbs indicate good bilateral symmetry, a characteristic often associated with enhanced performance and reduced risk of injury (Afonso et al., 2022).

Evidence suggests that the SLH test alone is insufficient to identify at-risk female athletes (Guild, Lininger & Warren, 2021). Therefore, in this study, we incorporated this test into a comprehensive battery of functional performance assessments of female athletes. SLH scores in our sample were above average, likely due to the intense training and physical demands experienced by our participants, many of whom were involved in high-energy sports (Guild, Lininger & Warren, 2021). These results may represent both their natural physical abilities and the sport-specific skills developed through regular practice.

The female Saudi participants in our study demonstrated moderate PAP values and lower performance on the LEFT, indicating an elevated injury risk. A slower rate of LEFT performance has been linked to a higher risk of injury, and the extended completion times in our sample may reflect reduced agility and functional capacity. The mean PAP values were significantly lower than the normative PAP values in female athletes (Sayers et al., 1999). The observed association between higher SLJ heights and faster LEFT completion underscores the interplay between explosive lower-body power and agility performance. The strong correlation between PAP and SLJ height further underscores the need for power-based strength training programs to enhance muscular force production and anaerobic capacity.

A moderate positive correlation was observed between the SLJ and SLH, indicating that improvements in one measure of explosive strength may predict improvements in the other. While beneficial across different athletic movements, this relationship is not perfectly linear, consistent with previous findings showing that increases in SLJ performance moderately influence SLH outcomes (Dominguez-Navarro et al., 2023). These results suggest that incorporating explosive strength exercises can enhance overall performance and reduce the risk of injury.

Theoretically, we propose a conceptual model linking injury prevention knowledge to motor performance adaptations. First, injury prevention knowledge acts as a cognitive resource that shapes athletes’ risk appraisal and the perceived value of protective behaviors, consistent with the health belief model and the theory of planned behavior (Ritchie, Van den Broucke & Van Hal, 2021). Second, this appraisal promotes favorable attitudes and stronger behavioral intentions, increasing engagement in preventive behaviors such as proper movement technique, controlled joint loading during drills, and neuromuscular conditioning. Third, these behaviors accumulate into neuromuscular adaptations, including improved joint position sense and cognitive–motor integration. This process reflects the declarative-to-procedural learning transition and Fitts and Posner’s model of skill acquisition, where explicit knowledge becomes automated movement through practice and feedback (DeKeyser, 2020; De Carvalho et al., 2025). However, this translation is task-dependent: it is more evident in open-skill tasks, such as multidirectional agility that requires perceptual-decision integration, and less influential in closed-skill, ballistic tasks, such as maximal jumping, which depend primarily on physiological factors. This prediction provides the theoretical basis for the differing associations between knowledge and agility- *versus* power-based outcomes examined in this study.

This study provides valuable insights into the relationship between knowledge, attitudes, and injury risk performance measures, emphasizing the critical role of education and structured training in optimizing athletic performance and preventing injuries in sports. The pattern of results emphasizes the three-stage theoretical model, in which declarative knowledge shapes behavioral dispositions, which are, in turn, consolidated into task-specific neuromuscular adaptations only in performance contexts that afford cognitive-motor integration. The LEFT demonstrated a stronger correlation with knowledge than PAP, SLH, and SLJ, presenting a central paradox: knowledge predicts agility (LEFT) but not power. This difference can be explained by the fundamentally different performance determinants of the tests. LEFT demands integrated cognitive-motor control, rapid anticipatory adjustments, efficient deceleration mechanics, and safe cutting strategies, all of which can be shaped by theoretical knowledge and biomechanical awareness (Avedesian et al., 2022). In this context, knowledge supports movement planning and optimizes trunk–lower limb alignment, reducing wasted motion and enhancing the efficiency of the directional change. In contrast, maximal power tasks, such as the SLJ and SLH, are predominantly closed-skill ballistic movements driven by neuromuscular capacity, fiber type distribution, and rate of force development, qualities that require targeted plyometric or resistance training to improve and are largely unaffected by declarative knowledge alone (Myer et al., 2006). From a motor learning perspective, agility tasks allow declarative knowledge to transition into procedural skills within an open-skill environment, whereas pure power outputs remain constrained by physiological adaptation thresholds (Hodges & Williams, 2012).

This distinction underscores the importance of task-specific cognitive-motor demands in the interpretation of functional outcomes. Knowledge of injury prevention can influence these processes by enhancing anticipatory decision-making, optimizing joint alignment, and promoting safer cutting mechanics, all of which can be consciously applied during open-skill, cognitively demanding tasks (Paul, Gabbett & Nassis, 2016; Sasaki et al., 2019; Avedesian et al., 2022; Robles-Palazón et al., 2024). In contrast, power-oriented measures, such as SLJ and SLH, are largely closed-skill, ballistic actions dominated by neuromuscular capacity, tendon stiffness, and rate of force development, which are improved primarily through targeted strength and plyometric training rather than declarative knowledge (Myer et al., 2006; Pedley et al., 2020). From a motor learning perspective, knowledge can be translated into performance when there is an opportunity for feedback, movement variability, and cognitive engagement, conditions present in agility tasks but minimal in maximal jump efforts (Forte et al., 2023). These differences highlight that the transfer of theoretical knowledge to performance is not uniform but depends on the degree to which the task demands cognitive-motor integration *versus* raw physical capacity. While knowledge alone may not enhance power-based performance, it remains essential for injury prevention, particularly when integrated into sport-specific training. For example, a knowledge-translation ACL injury prevention program improved shock absorption and knee stability by embedding biomechanical education within task-specific drills (Liao et al., 2025). The increased thigh angle and varus knee alignment observed in that study underscore the need to reinforce knowledge through dynamic, applied practice to produce meaningful changes in risk-related movement patterns (Liao et al., 2025).

Despite the high prevalence of positive injury prevention attitudes observed in this cohort, these attitudes were not associated with functional performance outcomes, suggesting that favorable perceptions alone may be insufficient to influence agility, balance, or power without corresponding behavioral implementation or structured training exposure. These findings align with previous research indicating that favorable perceptions do not always result in consistent adherence to preventive practices or measurable performance gains (Martinez et al., 2017). While both knowledge and attitudes are crucial, they must be linked to intentional and structured training programs to enhance performance. Education appeared to influence performance on the LEFT, a recognized indicator of injury risk, with knowledge explaining the variance in scores, highlighting the impact of educational awareness on functional agility.

This study sheds light on the key facilitators and challenges involved in implementing injury prevention programs among university athletes. Athlete adherence improves when programs are practical, performance-enhancing, and delivered by qualified professionals, consistent with evidence linking knowledgeable staff and structured guidance to higher compliance (Shamlaye, Tomšovský & Fulcher, 2020; Jimoh, 2024). Low coach involvement was a notable barrier, reflecting earlier findings that less experienced or overextended coaches may struggle to integrate prevention into training. Time constraints, reported by 71.67% of participants, were another significant barrier, aligning with literature citing limited training time as a common challenge (Dix et al., 2024).

Given that knowledge accounted for 14% of the variance in LEFT performance, integrating biomechanical education into agility-based drills may be a highly effective strategy for improving both movement efficiency and injury resilience. Such integration allows athletes to apply theoretical concepts, such as optimal deceleration angles, trunk control, and safe change-of-direction mechanics, directly within sport-specific contexts, which has been shown to improve functional movement quality and reduce lower limb injury risk (O’Brien & Finch, 2014; Liao et al., 2025). Given the reported time constraints, brief interventions like <10-minute Nordic hamstring routines or dynamic warm-ups may be more feasible for consistent implementation without disrupting training schedules. Short, evidence-based programs have shown high adherence and reductions in hamstring and knee injuries, even in tight training schedules (Soomro et al., 2016; Van Dyk, Behan & Whiteley, 2019). Tailoring such modules to the female university athlete context could maximize both feasibility and impact.

The differential findings across performance domains have direct implications for how practitioners structure training. For agility development, injury-prevention education should be embedded within the drills themselves rather than delivered as a separate classroom component: coaches can verbally cue biomechanical principles during change-of-direction exercises, use brief video review to reinforce correct mechanics, and assign knowledge-check tasks linked to agility warm-ups. This embedded approach leverages the open-skill, decision-making nature of agility tasks to accelerate the declarative-to-procedural knowledge transfer identified in this study. For power-based outcomes (jump height, anaerobic capacity), educational components are unlikely to substitute for progressive overload and plyometric conditioning; practitioners should therefore direct the majority of physical training time toward strength and power development without diverting it to knowledge delivery in that domain. In short, education and physical training are not competing resources but domain-specific tools: allocate cognitive instruction where task demands support its transfer, and physical load where they do not.

From a practical standpoint, the magnitude of the observed associations warrants explicit consideration. The correlation between knowledge and LEFT performance represents a moderate effect size that carries meaningful practical relevance. In the context of agility-based functional performance, a moderate effect implies that differences in injury prevention knowledge are associated with perceptible and clinically meaningful differences in movement efficiency. Furthermore, 14% of the variance in agility performance can be attributed to knowledge beyond training-related covariates. For coaches and practitioners, this finding suggests that targeted educational interventions on movement quality may yield tangible improvements in athletes’ functional agility over and above the effects of physical training alone. Given the relative low cost and logistical feasibility of educational components compared with structured physical training, even modest gains attributable to knowledge enhancement may represent high-value investments in athlete safety and performance optimization.

The significant positive relationship between knowledge of injury risk and LEFT performance underscores the importance of integrating injury risk awareness throughout all phases of the athlete’s training and development. At the foundational level, conditioning programs should incorporate comprehensive neuromuscular training protocols that address the biomechanical and physiological deficits associated with the risk of ACL injury. These protocols should include systematic training in balance, proprioception, and dynamic stability, delivered alongside educational components that explain the biomechanical risk factors contributing to non-contact ACL injuries. By pairing physical training with conceptual knowledge, athletes develop both the motor capacity and cognitive understanding necessary to prevent injuries during high-risk movements.

Systemic barriers also emerged in the form of unequal coaching practices and inconsistent administrative support. While some coaches prioritized injury prevention, others placed little emphasis on it, resulting in inconsistent applications across teams. This highlights the need for more standardized coach education and organizational support. Similar issues have been documented in previous studies, where a lack of institutional commitment and disparities in training quality have hindered the consistent adoption of best practices (Dix et al., 2024). Without unified support structures, even well-designed programs may fail in practice. Overall, injury mitigation strategies should be embedded within the existing culture and realities of university sports, supported in evidence-based practice, but adapted to time constraints, motivation level, and team dynamics. Enhancing coach education and ensuring administrative support are essential for creating an environment conducive to successful injury prevention.

While this study offers valuable insights into the knowledge, attitudes, and lower extremity performance of collegiate female athletes regarding injury prevention, it has several limitations must be considered. Although the questionnaire underwent content and face validity reviews, its self-reported nature presents a potential recall bias. Additionally, the absence of sport-specific stratification limits the ability to account for differences in injury risk profiles and prevention strategies between sports. Future research should consider stratifying participants by sport to allow for more precise and contextually relevant analysis, as this falls beyond the scope of the current study. Although the present study included participants from multiple sports, we acknowledge that sport-specific demands, injury mechanisms, and training protocols may differentially impact the relationship between knowledge of the risk of injury and LEFT performance. The heterogeneity of sports represented in our sample, although strengthening the generalizability to diverse athletic populations, may obscure sport-specific patterns that could have important implications for injury prevention and performance optimization. Future research should employ stratified or sport-specific analyses to determine whether the protective effect of knowledge of the risk of injury on LEFT performance is consistent across different sports.

The exclusive focus on female athletes restricts the generalizability of the results to male athletes or to the wider athletic population. Including participants of different genders and age groups in future investigations would offer a more comprehensive understanding of the knowledge, attitudes, and physical performance related to injury prevention across a broader athletic context. Finally, the cross-sectional design of this study precludes any determination of causal direction, and bidirectional pathways cannot be excluded. It is equally conceivable that the relationship is mutually reinforcing knowledge informs safer movement execution, which in turn supports physical development, and further motivates athletes to seek out and retain injury-prevention information. Disentangling these directional possibilities requires longitudinal or experimental designs in which knowledge is manipulated prior to performance assessment, and future studies should explicitly test both the knowledge-to-performance and performance-to-knowledge pathways to clarify the nature of this association. Future research should employ gender- and age-comparative designs to determine whether the knowledge–performance relationship generalizes across populations. Randomized controlled trials integrating biomechanical education into agility-based training are needed to establish causality, while longitudinal cohort studies would clarify whether knowledge acquisition predicts injury-preventive behaviors and whether behavioral change mediates the knowledge–injury outcome relationship.

## Conclusions

This study examined the relationship between injury prevention knowledge, attitudes, and selected lower extremity performance measures among female college athletes. Overall, the injury prevention knowledge was moderate. Notably, 38.3% of participants scored below 65%, indicating meaningful variability in knowledge levels and highlighting that a substantial proportion of athletes may possess a less-than-adequate understanding of injury prevention principles. These findings underscore the need for targeted, structured educational interventions.

Knowledge was significantly correlated with agility performance (LEFT) but not with power-based measures (PAP and SLH). This association is noteworthy, although the cross-sectional design precludes causal inference, and alternative explanations such as sport type and prior training exposure may account for this relationship. The stronger association with agility, a task requiring coordination and biomechanical control, compared to explosive power tasks, aligns with theoretical expectations regarding knowledge application in specific motor domains. Future research could employ longitudinal or experimental designs to investigate whether targeted biomechanical education enhances agility and injury resilience through improved movement efficiency. The SEBT results fell below the recommended 94% threshold across all directions, indicating balance deficits that may increase injury risk and emphasizing the need for targeted balance and proprioceptive training. Several key facilitators and barriers to the implementation of injury prevention programs were identified. Factors such as the presence of medical staff, athlete and coach education, access to quality equipment, and perceived performance benefits serve as strong motivators for adherence. Overall, this study underscores the need for a structured, multifaceted approach to injury prevention among female college athletes.

##  Supplemental Information

10.7717/peerj.21327/supp-1 Supplemental Information 1 Raw Dataset All raw data collected and analyzed in this study including participants’ demographic and all outcomes information.

10.7717/peerj.21327/supp-2 Supplemental Information 2 Codebook

10.7717/peerj.21327/supp-3 Supplemental Information 3 Attitude towards injury prevention of participants Responses are presented as frequencies and percentages for each Likert- scale category. The median score is provided to summarize the central tendency of responses for each item. Items assess athletes’ perceptions of injury risk, reporting behavior, attitudes toward playing while injured, and the perceived importance of injury prevention and medical evaluation.

## Abbreviations

SLJ: Countermovement Standing Long Jump
LEFT: Lower Extremity Functional Test
SLH: Single-Leg Hop
SEBT: Star Excursion Balance Test
Sec: Second
PAP: Peak Anaerobic Power
W: Watts

## Additional Information and Declarations

### Data Availability

The following information was supplied regarding data availability: The raw data are available in the Supplemental File.
