# Supplementary material for: Knowledge predicts agility but not power: the role of injury prevention awareness in the functional performance of female collegiate athletes
Source: PeerJ. 2026 May 22;14:e21327. doi: 10.7717/peerj.21327 (PMC13200619; doi:10.7717/peerj.21327)
Supplement: Supplemental Information 3 — Responses are presented as frequencies and percentages for each Likert- scale category. The median score is provided to summarize the central tendency of responses for each item. Items assess athletes’ perceptions of injury risk, reporting behavior, attitudes toward playing while injured, and the perceived importance of injury prevention and medical evaluation. [file peerj-14-21327-s003.docx]

| **Supplemental File**  **Table 1: Attitude towards injury prevention of participants** | | | | | | |
| --- | --- | --- | --- | --- | --- | --- |
| **Items** | **Never** | **Rarely** | **Sometimes** | **Often** | **Always** | **Median** |
|  | **N (%)** | **N (%)** | **N (%)** | **N (%)** | **N (%)** |  |
| As an athlete, I am at high risk of sustaining an injury. | 2 (3.33) | 8(13.33) | 26(43.33) | 19(31.67) | 5(8.33) | 2 |
| I will immediately inform my trainer, or any healthcare professional about my injury, even if it means I will have to sit out of the match. | 2 (3.33) | 4 (6.67) | 14(23.33) | 20(33.33) | 20(33.33) | 3 |
| I would play through any condition or injury for my team to win the game. | 4 (6.67) | 17 (28.33) | 15 (25) | 14 (23.33) | 10 (16.67) | 2 |
| I would inform other healthcare professionals about an injury, even if it meant missing a practice or two. | 4 (6.67) | 6 (10) | 14 (23.33) | 19 (31.67) | 17 (28.33) | 3 |
| I feel that it is important to be thoroughly evaluated by medical personnel after an injury to make sure I recover completely. | 3 (5) | 3 (5) | 9 (15) | 15 (25) | 30 (5) | 3.5 |
| During a championship game, you sustain an injury that hurts but does not affect your ability to play. Knowing that it would result in a more severe injury, I would inform the coach or athletic therapist about it. | 4 (6.67) | 10 (16.67) | 14 (23.33) | 20 (33.33) | 12 (20) | 3 |
| The motivation of the therapist affects the athletes' motivation to do injury prevention exercises? | 1 (1.67) | 2 (3.33) | 7 (11.67) | 17 928.33) | 33 (55) | 4 |
| **Items** | **Not important** | **Important Slightly** | **Important Moderately** | **Important** | **Very important** | **Median** |
| How important is it for you as an athlete to try to prevent injuries? | 1 (1.67) | 0 | 3 (5) | 13 (21.67) | 43 (71.67) | 4 |
| How important do you feel informed about injury prevention programs. | 3 (5) | 14 (23.33) | 29 (48.33) | 10 (16.67) | 4 (6.67) | 2 |
| How do you feel about injury prevention measures (e.g., stretching, specific warm-up exercise, taping)? | 1 (1.67) | 0 | 6 (10) | 21 (35) | 32 (53.33) | 4 |
